# Ethyl 2-(4-benzyl-3-methyl-6-oxo-1,6-dihydropyridazin-1-yl)acetate: crystal structure and Hirshfeld surface analysis

**DOI:** 10.1107/S205698901900241X

**Published:** 2019-02-22

**Authors:** Younes Zaoui, Youssef Ramli, Jamal Taoufik, Joel T. Mague, Mukesh M. Jotani, Edward R. T. Tiekink, M’hammed Ansar

**Affiliations:** a Laboratory of Medicinal Chemistry, Drug Sciences Research Center, Faculty of Medicine and Pharmacy, Mohammed V University, Rabat, Morocco; b Department of Chemistry, Tulane University, New Orleans, LA 70118, USA; c Department of Physics, Bhavan’s Sheth R. A. College of Science, Ahmedabad, Gujarat 380001, India; d Research Centre for Crystalline Materials, School of Science and Technology, Sunway University, 47500 Bandar Sunway, Selangor Darul Ehsan, Malaysia

**Keywords:** crystal structure, oxopyridazin­yl, ester, Hirshfeld surface analysis

## Abstract

The title compound, C_16_H_18_N_2_O_3_, is constructed about a central oxopyridazinyl ring (r.m.s. deviation = 0.0047 Å), which is connected to an ethyl­acetate group at the N atom closest to the carbonyl group, and benzyl and methyl groups second furthest and furthest from the carbonyl group, respectively. An approximately orthogonal relationship exists between the oxopyridazinyl ring and the best plane through the ethyl­acetate group [dihedral angle = 77.48 (3)°]; the latter lies to one side of the central plane [the N_r_—N_r_—C_m_—C_c_ (r = ring, m = methyl­ene, c = carbon­yl) torsion angle being 104.34 (9)°]. In the crystal, both H atoms of the N-bound methyl­ene group form methyl­ene-C—H⋯O(ring carbon­yl) or N(pyridazin­yl) inter­actions, resulting in the formation of a supra­molecular tape along the *a*-axis direction. The tapes are assembled into a three-dimensional architecture by methyl- and phenyl-C—H⋯O(ring carbon­yl) and phenyl-C—H⋯O(ester carbon­yl) inter­actions. The analysis of the calculated Hirshfeld surface indicates the dominance of H⋯H contacts to the overall surface (*i.e.* 52.2%). Reflecting other identified points of contact between mol­ecules noted above, O⋯H/H⋯O (23.3%), C⋯H/H⋯C (14.7%) and N⋯H/H⋯N (6.6%) contacts also make significant contributions to the surface.

## Abstract 2

In the title mol­ecule, the oxopyridazinyl ring is N-bound to an ethyl­acetate group with benzyl and methyl groups substituted at adjacent C atoms. In the mol­ecular packing, methyl­ene-C—H⋯O(ring carbon­yl) and N(pyridazin­yl) inter­actions result in the formation of a supra­molecular tape along the *a-*axis direction.

## Chemical context   

Pyridazin-3(2*H*)-ones are pyridazine derivatives, being constructed about a six-membered ring which contains two adjacent nitro­gen atoms, at positions one and two, and with a carbonyl group at position three. The inter­est in these nitro­gen-rich heterocyclic derivatives arises from the fact that they exhibit a number of promising pharmacological and biological activities. These include anti-oxidant (Khokra *et al.*, 2016 ▸), anti-bacterial and anti-fungal (Abiha *et al.* 2018 ▸), anti-cancer (Kamble *et al.* 2017 ▸), analgesic and anti-inflammatory (Ibrahim *et al.* 2017 ▸), anti-depressant (Boukharsa *et al.* 2016 ▸) and anti-ulcer activities (Yamada *et al.*, 1981 ▸). In addition, a number of pyridazinone derivatives have been reported to have potential as agrochemicals, for example as insecticides (Nauen & Bretschneider, 2002 ▸), acaricides (Igarashi & Sakamoto, 1994 ▸) and herbicides (Aza­ari *et al.*, 2016 ▸). Given the inter­est in this class of compound and the paucity in structural data (see *Database survey*), the crystal and mol­ecular structures of the the title pyridazin-3(2*H*)-one derivative, (I), has been undertaken along with an analysis of the calculated Hirshfeld surface in order to gain further insight into the mol­ecular packing.
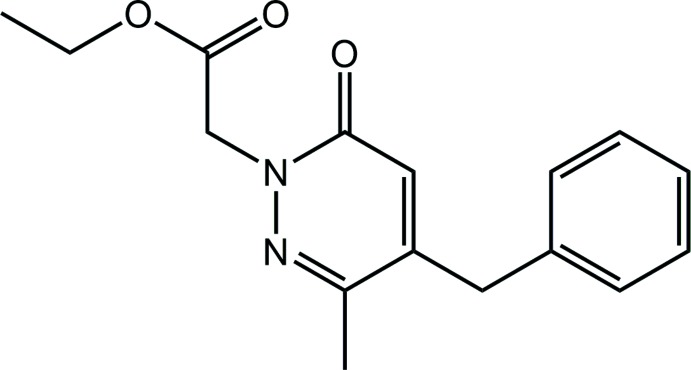


## Structural commentary   

**Figure 1:**
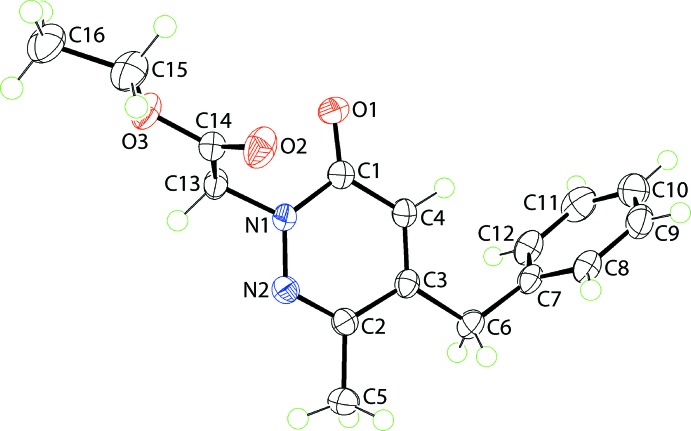
The mol­ecular structure of (I), showing the atom-labelling scheme and displacement ellipsoids at the 70% probability level.

The mol­ecular structure of (I), Fig. 1 ▸, comprises a central oxopyridazinyl ring connected to an ethyl­acetate group at the N1 atom, a methyl group at the C2 position and a benzyl residue at the C3 atom. The oxopyridazinyl ring is almost planar, having an r.m.s. deviation of 0.0047 Å for the ring atoms, with the maximum deviation from the ring being 0.0072 (6) Å for the C3 atom; the O1 atom lies 0.0260 (13) Å out of the plane in the same direction as the C3 atom. The ethyl acetate group is close to planar with the r.m.s. deviation for the O2,O3,C12–C16 atoms being 0.0476 Å [the maximum deviation from the least-squares plane is 0.0711 (7) Å for the O3 atom]. The dihedral angle between the two mentioned planes is 77.48 (3)°, indicating an approximately orthogonal relationship. The ethyl acetate group lies to one side of the central plane, as seen in the value of the N2—N1—C13—C14 torsion angle of 104.34 (9)°. The benzyl ring forms a dihedral angle of 76.94 (3)° with the central ring, also indicating an approximately orthogonal relationship but, in this case, the benzyl ring is bis­ected by the pseudo mirror plane passing through the oxopyridazinyl ring. Consistent with this, the pendant groups form a dihedral angle of 69.74 (3)°. Within the ester group, it is the carboxyl­ate-O3 atom that is directed away from the oxopyridazinyl ring so that the carbonyl-O1 and O2 atoms are proximate, at least to a first approximation.

## Supra­molecular features   

**Figure 2:**
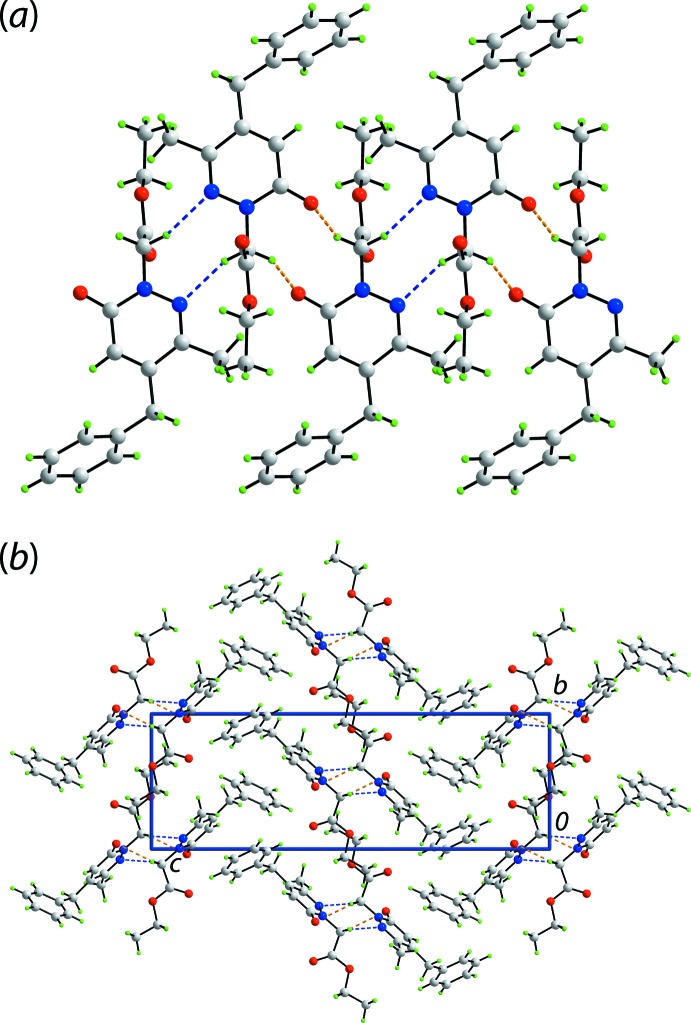
Supra­molecular association in the crystal of (I): (*a*) a view of the supra­molecular tape along the *a*-axis direction sustained by methyl­ene-C13—H⋯O1(ring carbon­yl) or N2(pyridazin­yl) inter­actions shown as orange and blue dashed lines, respectively, and (*b*) a view of the unit-cell contents shown in projection down the *a* axis.

The mol­ecular packing of (I) reveals a prominent role for the N1-bound methyl­ene group as each hydrogen atom of this residue participates in a methyl­ene-C13—H⋯O1(ring carbon­yl) or N2(pyridazin­yl) inter­action, Table 1 ▸, leading to ten-membered {⋯OCNCH}_2_ and eight-membered {⋯NNCH}_2_ synthons, respectively. The result is the formation of a supra­molecular tape orientated along the *a*-axis direction, Fig. 2 ▸(*a*). Globally, the tapes assemble into layers in the *ab* plane and these stack along the *c*-axis direction as shown in Fig. 2 ▸(*b*). Weak inter­actions contributing to the formation of the layers include methyl-C16—H⋯O1(ring carbon­yl) contacts (Table 2 ▸). Between layers are weak contacts of the type phenyl-C8, C9—H⋯O2(ester carbon­yl), phenyl-C10⋯O1(ring carbon­yl) and π–π between the oxopyridazinyl and phenyl ring [inter-centroid separation = 3.9573 (7) Å, angle of inclination = 15.00 (4)° for symmetry operation 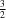 − *x*, 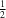 + *y*, 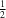 − *z*]. These inter­actions are discussed further in the section *Hirshfeld surface analysis*.

## Hirshfeld surface analysis   

**Figure 3:**
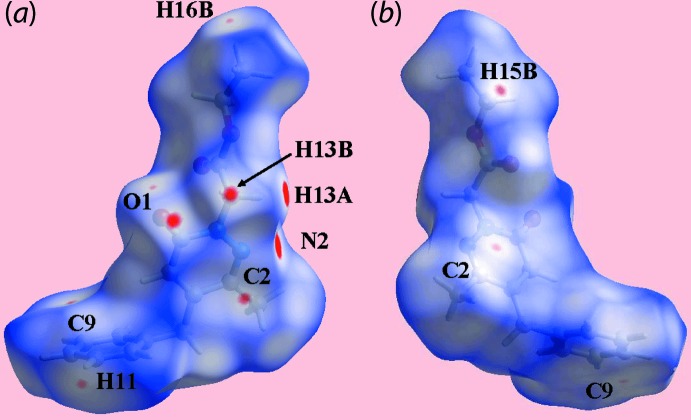
Two views of the Hirshfeld surface for (I) mapped over *d*_norm_ in the range −0.085 to +1.271 arbitrary units.

**Figure 4:**
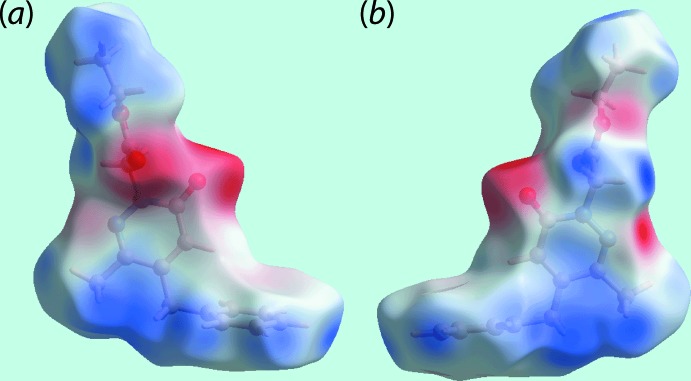
Two views of the Hirshfeld surface mapped over the electrostatic potential in the range −0.076 to +0.039 atomic units. The red and blue regions represent negative and positive electrostatic potentials, respectively.

**Figure 5:**
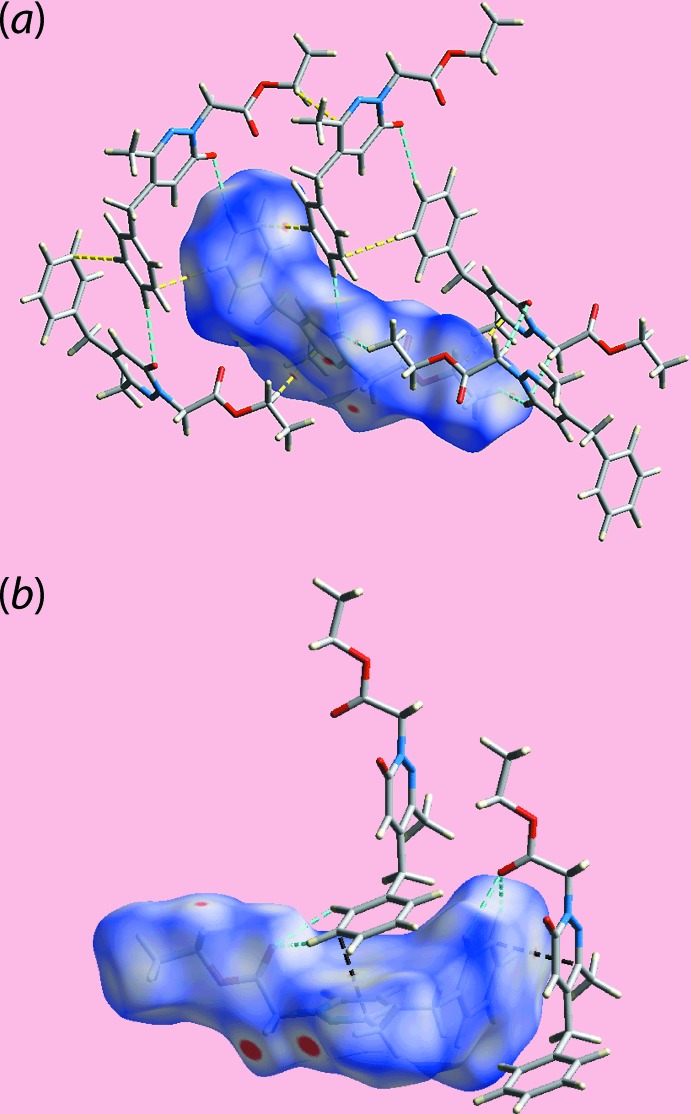
Two views of Hirshfeld surface mapped over *d*_norm_ in the range −0.085 to +1.271 arbitrary units showing significant short inter atomic O⋯H/H⋯O, C⋯H/H⋯C and C⋯C contacts by sky-blue, yellow and black dotted lines, respectively.

The Hirshfeld surfaces calculated for (I) were performed in accord with recent studies (Tan *et al.*, 2019 ▸) in order to provide complementary information on the influence of short inter­atomic contacts on the mol­ecular packing. On the Hirshfeld surfaces mapped over *d*_norm_ in Fig. 3 ▸(*a*), the C—H⋯N contact involving the methyl­ene-H13*A* and pyridazinyl-N2 atoms are represented as bright-red spots on the surface. The diminutive red spots appearing near the methyl­ene-H13*B* and carbonyl-O1 atoms indicate the weak C—H⋯O contact, Fig. 3 ▸(*a*) and (*b*). The intense blue and red regions corresponding to positive and negative electrostatic potentials on the Hirshfeld surfaces mapped over electrostatic potential in Fig. 4 ▸ also represent the donors and acceptors of the above inter­molecular inter­actions, respectively. The influence of the short inter­atomic O⋯H/H⋯O, C⋯H/H⋯C and C⋯C contacts, as summarized in Table 2 ▸, are viewed as the faint-red spots on the *d*_norm_-mapped Hirshfeld surfaces in Fig. 3 ▸. The environment of short inter­atomic O⋯H/H⋯O, C⋯H/H⋯C and C⋯C contacts about the reference mol­ecule within *d*_norm_ mapped Hirshfeld surface illustrating weak inter­molecular inter­actions are shown in the views of Fig. 5 ▸.

**Figure 6:**
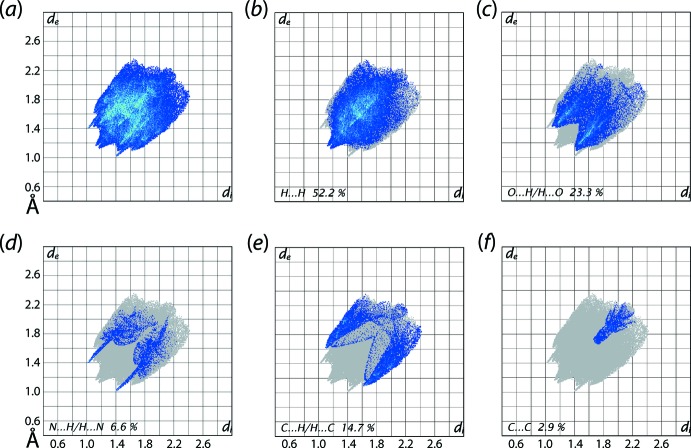
(*a*) The full two-dimensional fingerprint plot for (I) and (*b*)–(*f*) those delineated into H⋯H, O⋯H/H⋯O, N⋯H/H⋯N, C⋯H/H⋯C and C⋯C, contacts, respectively.

The overall two-dimensional fingerprint plot, Fig. 6 ▸(*a*), and those delineated into H⋯H, O⋯H/H⋯O, N⋯H/H⋯N and C⋯H/H⋯C and C⋯C contacts (McKinnon *et al.*, 2007 ▸) are illustrated in Fig. 6 ▸(*b*)–(*f*); the percentage contribution from different inter­atomic contacts to the Hirshfeld surfaces of (I) are summarized in Table 3 ▸. In the fingerprint plot delineated into H⋯H contacts shown in Fig. 6 ▸(*b*), having the greatest contribution, *i.e*. 52.2%, to the Hirshfeld surface, a pair of beak-shaped tips at *d*_e_ + *d*_i_ ∼2.3 Å reflect the short inter­atomic contact between the methyl-H5*C* and H16*C* atoms, Table 2 ▸. The fingerprint plot delineated into O⋯H/H⋯O contacts in Fig. 6 ▸(*c*) demonstrates two pairs of adjoining short tips at *d*_e_ + *d*_i_ ∼2.5 and 2.6 Å, together with the green aligned points in the central region, which are indicative of weak C—H⋯O contacts present in the crystal. The pair of long spikes at *d*_e_ + *d*_i_ ∼2.5 Å in the fingerprint plot delineated into N⋯H/H⋯N contacts of Fig. 6 ▸(*d*), are the result of a potential C—H⋯N inter­action involving the methyl­ene-C13—H13*A* and pyridazinyl-N2 atoms. The short inter­atomic C⋯H/H⋯C contacts as summarized in Table 2 ▸ are represented by a pair of forceps-like and parabolic tips a *d*_e_ + *d*_i_ ∼2.7 and 2.8 Å, respectively in Fig. 6 ▸(*e*). The presence of a weak π–π contact between the oxopyridazinyl and phenyl rings is reflected in the thick arrow-like tip at *d*_e_ + *d*_i_ ∼3.4 Å in the fingerprint plot delineated into C⋯C contacts of Fig. 6 ▸(*f*), specifically the short inter­atomic C2⋯C9 contact, Table 2 ▸, and the small but notable, *i.e*. 2.3%, contribution from C⋯N/N⋯C contacts to the Hirshfeld surface.

## Database survey   

**Figure 7:**
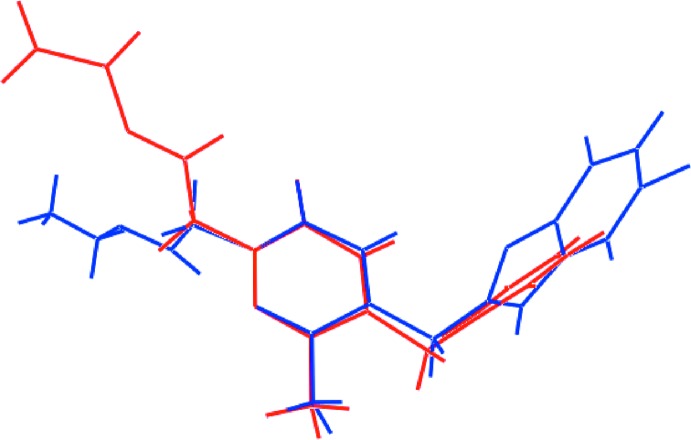
Overlay diagram of (I) (red image) and literature analogue (II) (blue). The mol­ecules have been aligned so the NO_2_ atoms of the central ring are coincident.

The most closely related structure to (I) in the crystallographic literature is compound (II) whereby the benzyl group of (I) is substituted by a (5-chloro-1-benzo­furan-2-yl)meth­yl) group (Aydın *et al.*, 2007 ▸). The structure of (II) presents the same features as for (I) but, with the ester-carbonyl atom directed away from the ring carbonyl group as highlighted in the overlay diagram of Fig. 7 ▸.

## Synthesis and crystallization   

A mixture of 3-benzyl­idene-4-oxo­penta­noic acid (0.05 mol) and hydrazine hydrate (0.1 mol) in ethanol (100 ml) was refluxed for 2 h. The precipitate formed was filtered off and recrystallized from acetone to obtain the 5-benzyl-6-methyl­pyridazin-3(2*H*)-one precursor. To this pyridazine (0.05 mol) was added potassium carbonate (0.1 mmol), tetra­butyl­ammonium bromide (0.01 mmol) and 2-ethyl bromo­acetate (0.1 mol) in di­methyl­formamide (20 ml). The mixture was stirred for 24 h at room temperature. At the end of the reaction, the solution was filtered and the solvent evaporated under reduced pressure. The residue was washed with water and methyl­enechloride. The solvent was removed and colourless blocks of (I) were obtained by recrystallization of the product from its acetone solution.

## Refinement details   

Crystal data, data collection and structure refinement details are summarized in Table 4 ▸. The carbon-bound H atoms were placed in calculated positions (C—H = 0.95–0.99 Å) and included in the refinement in the riding model approximation, with *U*_iso_(H) set to 1.2–1.5*U*_eq_(C).

## Supplementary Material

Crystal structure: contains datablock(s) I, global. DOI: 10.1107/S205698901900241X/hb7802sup1.cif

Structure factors: contains datablock(s) I. DOI: 10.1107/S205698901900241X/hb7802Isup2.hkl

Supporting information file. DOI: 10.1107/S205698901900241X/hb7802Isup3.cml

CCDC reference: 1897511

Additional supporting information:  crystallographic information; 3D view; checkCIF report

## Figures and Tables

**Table 1:** Hydrogen-bond geometry (Å, °)

*D*—H⋯*A*	*D*—H	H⋯*A*	*D*⋯*A*	*D*—H⋯*A*
C13—H13*A*⋯N2^i^	0.99	2.51	3.4704 (13)	165
C13—H13*B*⋯O1^ii^	0.99	2.59	3.4281 (13)	143

Symmetry codes: (i) 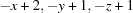; (ii) 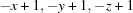.

**Table 2:** Summary of short inter­atomic contacts (Å) in (I)

Contact	Distance	Symmetry operation
H5*C*⋯H16*C*	2.29	2 − *x*, 1 − *y*, 1 − *z*
O1⋯H10	2.58	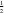 − *x*, 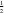 + *y*, 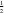 − *z*
O1⋯H16*B*	2.55	1 − *x*, 2 − *y*, 1 − *z*
O2⋯H8	2.63	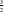 − *x*, 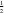 + *y*, 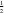 − *z*
O2⋯H9	2.63	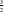 − *x*, 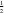 + *y*, 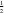 − *z*
C2⋯H1*B*5	2.71	*x*, −1 + *y*, *z*
C9⋯H11	2.73	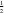 − *x*, 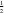 + *y*, 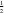 − *z*
C10⋯H5*B*	2.81	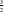 − *x*, − 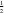 + *y*, 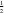 − *z*
C2⋯C9	3.3683 (14)	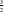 − *x*, 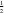 + *y*, 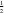 − *z*

**Table 3:** Percentage contributions of inter­atomic contacts to the Hirshfeld surface for (I)

Contact	Percentage contribution
H⋯H	52.2
O⋯H/H⋯O	23.3
C⋯H/H⋯C	14.7
N⋯H/H⋯N	6.6
C⋯C	2.9
C⋯N/N⋯C	0.3

**Table 4:** Experimental details

Crystal data	
Chemical formula	C_16_H_18_N_2_O_3_
*M*_r_	286.32
Crystal system, space group	Monoclinic, *P*2_1_/*n*
Temperature (K)	120
*a*, *b*, *c* (Å)	7.4069 (9), 8.1959 (10), 24.133 (3)
β (°)	90.295 (2)
*V* (Å^3^)	1465.0 (3)
*Z*	4
Radiation type	Mo *K*α
μ (mm^−1^)	0.09
Crystal size (mm)	0.37 × 0.29 × 0.24

Data collection	
Diffractometer	Bruker *SMART* *APEX* CCD
Absorption correction	Multi-scan (*SADABS*; Krause *et al.*, 2015 ▸)
*T*_min_, *T*_max_	0.91, 0.98
No. of measured, independent and observed [*I* > 2σ(*I*)] reflections	27503, 3966, 3354
*R*_int_	0.028
(sin θ/λ)_max_ (Å^−1^)	0.688

Refinement	
*R*[*F* ^2^ > 2σ(*F* ^2^)], *wR*(*F* ^2^), *S*	0.041, 0.120, 1.09
No. of reflections	3966
No. of parameters	192
H-atom treatment	H-atom parameters constrained
Δρ_max_, Δρ_min_ (e Å^−3^)	0.43, −0.16

Computer programs: *APEX3* and *SAINT* (Bruker, 2016 ▸), *SHELXT* (Sheldrick, 2015*a* ▸), *SHELXL2014* (Sheldrick, 2015*b* ▸), *ORTEP-3 for Windows* (Farrugia, 2012 ▸), *DIAMOND* (Brandenburg, 2006 ▸) and *publCIF* (Westrip, 2010 ▸).

## supplementary crystallographic information

### Crystal data

**Table:**

C_16_H_18_N_2_O_3_	*F*(000) = 608
*M**_r_* = 286.32	*D*_x_ = 1.298 Mg m^−^^3^
Monoclinic, *P*2_1_/*n*	Mo *K*α radiation, λ = 0.71073 Å
*a* = 7.4069 (9) Å	Cell parameters from 9950 reflections
*b* = 8.1959 (10) Å	θ = 2.6–29.2°
*c* = 24.133 (3) Å	µ = 0.09 mm^−^^1^
β = 90.295 (2)°	*T* = 120 K
*V* = 1465.0 (3) Å^3^	Block, colourless
*Z* = 4	0.37 × 0.29 × 0.24 mm

### Data collection

**Table:**

Bruker SMART APEX CCD diffractometer	3966 independent reflections
Graphite monochromator	3354 reflections with *I* > 2σ(*I*)
Detector resolution: 8.3333 pixels mm^-1^	*R*_int_ = 0.028
φ and ω scans	θ_max_ = 29.3°, θ_min_ = 1.7°
Absorption correction: multi-scan (*SADABS*; Krause *et al.*, 2015)	*h* = −10→10
*T*_min_ = 0.91, *T*_max_ = 0.98	*k* = −11→11
27503 measured reflections	*l* = −32→32

### Refinement

**Table:**

Refinement on *F*^2^	Primary atom site location: dual
Least-squares matrix: full	Hydrogen site location: inferred from neighbouring sites
*R*[*F*^2^ > 2σ(*F*^2^)] = 0.041	H-atom parameters constrained
*wR*(*F*^2^) = 0.120	*w* = 1/[σ^2^(*F*_o_^2^) + (0.0785*P*)^2^ + 0.123*P*] where *P* = (*F*_o_^2^ + 2*F*_c_^2^)/3
*S* = 1.09	(Δ/σ)_max_ < 0.001
3966 reflections	Δρ_max_ = 0.43 e Å^−^^3^
192 parameters	Δρ_min_ = −0.15 e Å^−^^3^
0 restraints	

### Special details

**Table:**

Experimental. The diffraction data were obtained from 3 sets of 400 frames, each of width 0.5° in ω, colllected at φ = 0.00, 90.00 and 180.00° and 2 sets of 800 frames, each of width 0.45° in φ, collected at ω = –30.00 and 210.00°. The scan time was 15 sec/frame.
Geometry. All esds (except the esd in the dihedral angle between two l.s. planes) are estimated using the full covariance matrix. The cell esds are taken into account individually in the estimation of esds in distances, angles and torsion angles; correlations between esds in cell parameters are only used when they are defined by crystal symmetry. An approximate (isotropic) treatment of cell esds is used for estimating esds involving l.s. planes.

### Fractional atomic coordinates and isotropic or equivalent isotropic displacement parameters (Å^2^)

**Table:**

	*x*	*y*	*z*	*U*_iso_*/*U*_eq_	
O1	0.46272 (9)	0.53825 (9)	0.41137 (3)	0.02901 (19)	
O2	0.76767 (11)	0.83037 (9)	0.40668 (3)	0.02903 (18)	
O3	0.73810 (9)	0.88740 (8)	0.49738 (3)	0.02266 (17)	
N1	0.76025 (10)	0.49470 (9)	0.42943 (3)	0.01813 (17)	
N2	0.92321 (10)	0.42213 (9)	0.42082 (3)	0.01830 (17)	
C1	0.60366 (12)	0.46663 (11)	0.39934 (4)	0.02018 (19)	
C2	0.93494 (12)	0.31446 (11)	0.38109 (4)	0.01719 (18)	
C3	0.78234 (12)	0.27085 (10)	0.34652 (4)	0.01715 (18)	
C4	0.62344 (12)	0.34749 (11)	0.35575 (4)	0.01990 (19)	
H4	0.5223	0.3222	0.3329	0.024*	
C5	1.11600 (13)	0.23628 (12)	0.37348 (4)	0.0240 (2)	
H5A	1.2046	0.2896	0.3977	0.036*	
H5B	1.1535	0.2479	0.3348	0.036*	
H5C	1.1083	0.1202	0.3829	0.036*	
C6	0.80584 (13)	0.14014 (11)	0.30267 (4)	0.0217 (2)	
H6A	0.9049	0.1729	0.2776	0.026*	
H6B	0.8421	0.0371	0.3210	0.026*	
C7	0.63798 (13)	0.10948 (11)	0.26856 (4)	0.01965 (19)	
C8	0.61870 (13)	0.18014 (11)	0.21636 (4)	0.0222 (2)	
H8	0.7153	0.2417	0.2012	0.027*	
C9	0.45975 (15)	0.16155 (12)	0.18614 (4)	0.0277 (2)	
H9	0.4485	0.2100	0.1505	0.033*	
C10	0.31775 (15)	0.07256 (13)	0.20780 (5)	0.0314 (2)	
H10	0.2081	0.0621	0.1875	0.038*	
C11	0.33642 (14)	−0.00130 (13)	0.25926 (5)	0.0306 (2)	
H11	0.2401	−0.0642	0.2739	0.037*	
C12	0.49596 (14)	0.01645 (12)	0.28948 (4)	0.0253 (2)	
H12	0.5082	−0.0351	0.3246	0.030*	
C13	0.75566 (13)	0.61201 (11)	0.47460 (4)	0.01904 (19)	
H13A	0.8627	0.5955	0.4987	0.023*	
H13B	0.6466	0.5924	0.4972	0.023*	
C14	0.75378 (12)	0.78657 (11)	0.45400 (4)	0.01866 (19)	
C15	0.74349 (16)	1.06125 (11)	0.48474 (4)	0.0277 (2)	
H15A	0.6350	1.0933	0.4632	0.033*	
H15B	0.8518	1.0871	0.4625	0.033*	
C16	0.74939 (16)	1.15128 (12)	0.53893 (5)	0.0305 (2)	
H16A	0.6443	1.1210	0.5612	0.046*	
H16B	0.7475	1.2690	0.5319	0.046*	
H16C	0.8602	1.1225	0.5590	0.046*	

### Atomic displacement parameters (Å^2^)

**Table:**

	*U*^11^	*U*^22^	*U*^33^	*U*^12^	*U*^13^	*U*^23^
O1	0.0213 (3)	0.0338 (4)	0.0318 (4)	0.0072 (3)	−0.0028 (3)	−0.0136 (3)
O2	0.0441 (5)	0.0257 (4)	0.0173 (4)	0.0028 (3)	0.0022 (3)	0.0014 (3)
O3	0.0351 (4)	0.0156 (3)	0.0173 (3)	0.0007 (3)	0.0015 (3)	−0.0014 (2)
N1	0.0196 (4)	0.0183 (4)	0.0164 (4)	0.0021 (3)	−0.0026 (3)	−0.0039 (3)
N2	0.0188 (4)	0.0188 (3)	0.0173 (4)	0.0016 (3)	−0.0010 (3)	0.0014 (3)
C1	0.0195 (4)	0.0211 (4)	0.0200 (4)	0.0010 (3)	−0.0018 (3)	−0.0034 (3)
C2	0.0192 (4)	0.0172 (4)	0.0151 (4)	0.0014 (3)	−0.0004 (3)	0.0027 (3)
C3	0.0218 (4)	0.0151 (4)	0.0145 (4)	−0.0001 (3)	−0.0001 (3)	0.0003 (3)
C4	0.0203 (4)	0.0204 (4)	0.0190 (4)	0.0002 (3)	−0.0033 (3)	−0.0043 (3)
C5	0.0208 (4)	0.0282 (5)	0.0229 (5)	0.0064 (4)	−0.0006 (4)	−0.0002 (4)
C6	0.0252 (5)	0.0194 (4)	0.0206 (5)	0.0032 (3)	−0.0001 (4)	−0.0052 (3)
C7	0.0253 (5)	0.0162 (4)	0.0175 (4)	0.0008 (3)	0.0012 (3)	−0.0045 (3)
C8	0.0290 (5)	0.0188 (4)	0.0188 (4)	−0.0001 (3)	0.0031 (4)	−0.0022 (3)
C9	0.0366 (6)	0.0256 (5)	0.0208 (5)	0.0050 (4)	−0.0034 (4)	−0.0053 (4)
C10	0.0298 (5)	0.0295 (5)	0.0350 (6)	0.0000 (4)	−0.0071 (4)	−0.0138 (4)
C11	0.0304 (5)	0.0245 (5)	0.0370 (6)	−0.0081 (4)	0.0061 (4)	−0.0089 (4)
C12	0.0346 (5)	0.0202 (4)	0.0212 (5)	−0.0032 (4)	0.0047 (4)	−0.0022 (3)
C13	0.0244 (4)	0.0181 (4)	0.0146 (4)	0.0004 (3)	−0.0019 (3)	−0.0026 (3)
C14	0.0187 (4)	0.0201 (4)	0.0172 (4)	0.0004 (3)	−0.0009 (3)	−0.0023 (3)
C15	0.0412 (6)	0.0157 (4)	0.0261 (5)	0.0018 (4)	0.0013 (4)	0.0011 (4)
C16	0.0397 (6)	0.0185 (5)	0.0332 (6)	0.0025 (4)	0.0003 (4)	−0.0054 (4)

### Geometric parameters (Å, º)

**Table:**

O1—C1	1.2336 (11)	C7—C8	1.3932 (13)
O2—C14	1.2020 (11)	C7—C12	1.3958 (13)
O3—C14	1.3392 (10)	C8—C9	1.3901 (14)
O3—C15	1.4577 (11)	C8—H8	0.9500
N1—N2	1.3625 (10)	C9—C10	1.3846 (16)
N1—C1	1.3845 (12)	C9—H9	0.9500
N1—C13	1.4541 (11)	C10—C11	1.3878 (16)
N2—C2	1.3063 (11)	C10—H10	0.9500
C1—C4	1.4434 (12)	C11—C12	1.3930 (15)
C2—C3	1.4463 (12)	C11—H11	0.9500
C2—C5	1.4985 (12)	C12—H12	0.9500
C3—C4	1.3535 (13)	C13—C14	1.5145 (12)
C3—C6	1.5164 (12)	C13—H13A	0.9900
C4—H4	0.9500	C13—H13B	0.9900
C5—H5A	0.9800	C15—C16	1.5020 (14)
C5—H5B	0.9800	C15—H15A	0.9900
C5—H5C	0.9800	C15—H15B	0.9900
C6—C7	1.5088 (13)	C16—H16A	0.9800
C6—H6A	0.9900	C16—H16B	0.9800
C6—H6B	0.9900	C16—H16C	0.9800
			
C14—O3—C15	115.91 (7)	C7—C8—H8	119.6
N2—N1—C1	126.01 (7)	C10—C9—C8	120.17 (10)
N2—N1—C13	115.28 (7)	C10—C9—H9	119.9
C1—N1—C13	118.70 (7)	C8—C9—H9	119.9
C2—N2—N1	117.97 (7)	C9—C10—C11	119.69 (10)
O1—C1—N1	120.34 (8)	C9—C10—H10	120.2
O1—C1—C4	125.64 (8)	C11—C10—H10	120.2
N1—C1—C4	114.00 (8)	C10—C11—C12	120.21 (10)
N2—C2—C3	122.39 (8)	C10—C11—H11	119.9
N2—C2—C5	116.22 (8)	C12—C11—H11	119.9
C3—C2—C5	121.39 (8)	C11—C12—C7	120.44 (9)
C4—C3—C2	117.90 (8)	C11—C12—H12	119.8
C4—C3—C6	123.08 (8)	C7—C12—H12	119.8
C2—C3—C6	119.01 (8)	N1—C13—C14	112.26 (7)
C3—C4—C1	121.70 (8)	N1—C13—H13A	109.2
C3—C4—H4	119.1	C14—C13—H13A	109.2
C1—C4—H4	119.1	N1—C13—H13B	109.2
C2—C5—H5A	109.5	C14—C13—H13B	109.2
C2—C5—H5B	109.5	H13A—C13—H13B	107.9
H5A—C5—H5B	109.5	O2—C14—O3	124.51 (9)
C2—C5—H5C	109.5	O2—C14—C13	126.38 (8)
H5A—C5—H5C	109.5	O3—C14—C13	109.09 (7)
H5B—C5—H5C	109.5	O3—C15—C16	107.38 (8)
C7—C6—C3	113.65 (7)	O3—C15—H15A	110.2
C7—C6—H6A	108.8	C16—C15—H15A	110.2
C3—C6—H6A	108.8	O3—C15—H15B	110.2
C7—C6—H6B	108.8	C16—C15—H15B	110.2
C3—C6—H6B	108.8	H15A—C15—H15B	108.5
H6A—C6—H6B	107.7	C15—C16—H16A	109.5
C8—C7—C12	118.70 (9)	C15—C16—H16B	109.5
C8—C7—C6	120.30 (8)	H16A—C16—H16B	109.5
C12—C7—C6	120.94 (9)	C15—C16—H16C	109.5
C9—C8—C7	120.76 (9)	H16A—C16—H16C	109.5
C9—C8—H8	119.6	H16B—C16—H16C	109.5
			
C1—N1—N2—C2	−0.81 (13)	C3—C6—C7—C8	98.97 (10)
C13—N1—N2—C2	179.38 (7)	C3—C6—C7—C12	−78.09 (11)
N2—N1—C1—O1	179.08 (9)	C12—C7—C8—C9	1.41 (13)
C13—N1—C1—O1	−1.11 (13)	C6—C7—C8—C9	−175.72 (8)
N2—N1—C1—C4	0.48 (13)	C7—C8—C9—C10	0.24 (14)
C13—N1—C1—C4	−179.71 (8)	C8—C9—C10—C11	−1.55 (15)
N1—N2—C2—C3	0.03 (12)	C9—C10—C11—C12	1.21 (15)
N1—N2—C2—C5	−179.33 (8)	C10—C11—C12—C7	0.46 (15)
N2—C2—C3—C4	1.02 (13)	C8—C7—C12—C11	−1.75 (14)
C5—C2—C3—C4	−179.66 (8)	C6—C7—C12—C11	175.35 (9)
N2—C2—C3—C6	−177.78 (8)	N2—N1—C13—C14	104.34 (9)
C5—C2—C3—C6	1.55 (12)	C1—N1—C13—C14	−75.50 (10)
C2—C3—C4—C1	−1.34 (13)	C15—O3—C14—O2	−1.63 (13)
C6—C3—C4—C1	177.40 (8)	C15—O3—C14—C13	176.99 (8)
O1—C1—C4—C3	−177.86 (9)	N1—C13—C14—O2	−5.26 (14)
N1—C1—C4—C3	0.65 (13)	N1—C13—C14—O3	176.15 (7)
C4—C3—C6—C7	2.78 (13)	C14—O3—C15—C16	−172.44 (8)
C2—C3—C6—C7	−178.49 (8)		

### Hydrogen-bond geometry (Å, º)

**Table:**

*D*—H···*A*	*D*—H	H···*A*	*D*···*A*	*D*—H···*A*
C13—H13*A*···N2^i^	0.99	2.51	3.4704 (13)	165
C13—H13*B*···O1^ii^	0.99	2.59	3.4281 (13)	143

Symmetry codes: (i) −*x*+2, −*y*+1, −*z*+1; (ii) −*x*+1, −*y*+1, −*z*+1.
